# Supplementary material for: The relationship between anaemia and the use of treated bed nets among pregnant and non-pregnant women in Ghana
Source: PLoS One. 2024 May 2;19(5):e0300431. doi: 10.1371/journal.pone.0300431 (PMC11065244; doi:10.1371/journal.pone.0300431)
Supplement: S2 File — (ZIP) [file pone.0300431.s002.zip › S2_File.pdf]

Supplementary Information on the Methodology

Methods

The Curie-Weiss Hamiltonian

For a set of coded decisions, $\delta = (\delta_1, \delta_2, \cdots, \delta_D)$, the Curie-Weiss Hamiltonian at inverse temperature $K_{z,n}$ and an external magnetic field p_z are defined as follows:

$$H_D(\delta) = \frac{1}{2D} \sum_{z,n=1}^{D} K_{z,n} \delta_z \delta_n + \sum_{z=1}^{D} p_z \delta_z \tag{0.1}$$

where

$$\delta_z = \begin{cases} +1, & \text{if individual } z \text{ sleeps in TBN,} \\ -1, & \text{if individual, } z \text{ does not sleep in TBN .} \end{cases}$$

The Curie-Weiss Hamiltonian is divided into two parts: the interaction component, $K_{z,n}$, which represents the interaction strength between individuals z and n, and the external field of influence, p_z. If $K_{z,n}$ is positive, it indicates that conformity is rewarded, if $K_{z,n}$ is negative, it indicates that conformity is not rewarded. Spins pointing up are favoured when the external field p_z is directed upwards, while spins pointing down are favoured when the field is directed downwards [1].

As a benchmark model, the Multipopulation Curie-Weiss model for discrete choice with social interaction will be used in this work.

Multipopulation Curie-Weiss Model

Our main premise is that women with similar characteristics exhibit similar behaviour, whereas women with diverse attributes display diverse behaviours. This premise plays a crucial role in redefining the parameters of the Curie-Weiss Hamiltonian as shown in equation (0.1). Consequently, our primary objective is to determine a suitable parameterization for the interaction coefficient $K_{z,n}$ and develop a systematic approach for estimating the model's parameters using data. In line with our discrete choice model, each individual z is assigned a set of w characteristics:

$$\varphi_i = \left(\varphi_z^1, \varphi_z^2, \cdots, \varphi_z^w\right), \text{with } \varphi_z^j \in \{0, 1\}. \tag{0.2}$$

Consider the example where the parameters of interest are Pregnancy status φ_z^1 and anaemia status φ_z^2 with

$$\varphi_z^1 = \begin{cases} 1, & \text{if woman } z \text{ is pregnant} \\ 0, & \text{if woman } z \text{ is non-pregnant} \end{cases} \tag{0.3}$$

and

$$\varphi_z^2 = \begin{cases} 1, & \text{if woman } z \text{ is anaemic} \\ 0, & \text{if woman } z \text{ is non anaemic.} \end{cases} \tag{0.4}$$

As a result, the D-dimensional population is segmented into 2^w non-overlapping divisions. Let V_{D_a} represent the women in the partition $a = 1, \cdots, 2^w$ and $|V_{D_a}| = D_a$. Therefore $D = D_1 + D_2 + \cdots + D_{2^w}$. Let $V_D = \{1, 2, \cdots, D\}$ with $V_{D_a} \cap V_{D_b} = \emptyset$, for $a \neq b$. We also assume that, in each case, $a = 1, \cdots, 2^w$:

$$\gamma_D^a = \frac{D_a}{D} \quad \text{and} \quad \gamma^g = \lim_{D \to \infty} \gamma_D^a. \tag{0.5}$$

Because of our previous assumption, all women in the partition a have the identical private incentive p_a, and $K_{z,n} = K_{a,b}$ for every $z \in V_{D_a}$ and $n \in V_{D_b}$. With the preceding assumption and (0.1), $H_D(\delta)$ is reduced to 0.6.

$$H_D(\delta) = D \sum_{a=1}^{2^w} n_D^a P_{D,a} \tag{0.6}$$

where

$$n_D^a = \frac{1}{D_a} \sum_{s \in V_{D_a}} \gamma_s \text{ and } P_{D,a} = \sum_{b=1}^{2^w} \frac{\bar{K_{a,b}}}{2} \gamma_D^a \gamma_D^b n_D^b + \bar{p}_a \gamma_D^a \tag{0.7}$$

n_D^a is the average choice made by the partition a members. In our approach, individual alternatives have been substituted with communal possibilities. $H_D(\delta)$ yields the total degree of satisfaction for the entire population.

In (0.1), the equilibrium condition x_D coupled to the Hamiltonian $H_D(\delta)$ is defined by

$$x_D(\delta) = \frac{e^{H_D(\delta)} c_D}{r_D}, \text{ for } \delta \in \Omega_D \tag{0.8}$$

where c_D is the corresponding product measure on $\Omega_D = \{-1, 1\}^D$ and

$$r_D = \sum_{\hat{\delta} \in \Omega_D} e^{H_D(\hat{\delta})} c_D(\hat{\delta}) = \int_{[-1,1]^d} e^{DF_D(n_D)} dQ_D(n) \tag{0.9}$$

is the partition function and Q_D is the vector of averages $n_D = \left(n_D^1, \cdots, n_D^d\right) \in [-1, 1]^d$ under c_D and let $d = 2^w$. Note that

$$\lim_{D \to \infty} F_D(n) = F(n) \tag{0.10}$$

where

$$F_D(n_D) = \sum_{a=1}^{d} \sum_{b=1}^{d} \frac{\bar{K_{a,b}}}{2} \gamma_D^a \gamma_D^b n_D^a n_D^b + \sum_{a=1}^{d} \bar{p}_a \gamma_D^a n_D^a \tag{0.11}$$

and

$$F(n) = \sum_{a=1}^{d} \sum_{b=1}^{d} \frac{\bar{K_{a,b}}}{2} \gamma^a \gamma^b n^a n^b + \sum_{a=1}^{d} \bar{p}_a \gamma^a n^a. \tag{0.12}$$

Next, let

$$\gamma = \left(\gamma^1, \cdots, \gamma^d\right), \ \bar{p} = \left(\bar{p}^1, \cdots, \bar{p}^d\right) \in \mathbb{R}, \text{ and } \bar{K} = \left(\bar{K}_{a,b}\right)_{1 \leq a,b \leq d}. \tag{0.13}$$

The model's pressure function is given by

$$\mathbb{P}_D = \frac{1}{D} \ln(r_D) \tag{0.14}$$

From (0.14) the thermodynamic limit is given by

$$\mathbb{P}(\gamma, \bar{p}, \bar{K}) = \lim_{D \to \infty} \frac{1}{D} \ln(r_D) \tag{0.15}$$

Theorem 0.1 *The limiting pressure accepts the following variational formulation for any choice of the parameters $\gamma, \bar{p}$, and $\bar{K}$*

$$\mathbb{P}(\gamma, \bar{p}, \bar{K}) = \sup_{n \in [-1,1]^d} \left[F(n) - \sum_{a=1}^{d} \gamma^a H(n^a) \right], \tag{0.16}$$

where

$$H(n^a) = \frac{1+n^a}{2} \ln(1+n^a) + \frac{1-n^a}{2} \ln(1-n^a) \tag{0.17}$$

and the function $F(n)$ is defined in (0.12). n^a's maximizes (0.16) and fulfils the self-consistency equations.

$$n^a = \tanh(U_a),\ g = 1, 2, 3, \cdots, 2^w \tag{0.18}$$

where

$$U_a = \sum_{b=1}^{d} \frac{\bar{K_{a,b}}}{2} \gamma^a \gamma^b n^b + \bar{p}_a \gamma^a. \tag{0.19}$$

In this study, we would look at two attributes: pregnancy status (pregnant or non-pregnant) and anaemia (anaemic or non-anaemic), each with two alternative values, for a total of four divisions. Let us parameterize $K_{a,b} = \frac{\bar{K_{a,b}}}{2} \gamma^a \gamma^b$ and $p_a = \gamma^a \bar{p_a}$. Additionally, we can express the possibility that the ith woman in group a will select $\delta_i \in \{-1, +1\}$ to be given by

$$p(\delta_i) = \frac{e^{\delta_i U_a}}{e^{U_a} + e^{-U_a}}. \tag{0.20}$$

As we can see, the expected value of the ith individual's selection is given by

$$\mathbb{E}(\delta_i) = \frac{e^{U_a}}{e^{U_a} + e^{-U_a}} - \frac{e^{-U_a}}{e^{U_a} + e^{-U_a}} = \tanh(U_a). \tag{0.21}$$

According to the self-consistency equations (0.18), for any $a = 1, 2, \cdots, d$,

$$n^a = \mathbb{E}(\delta_i) = \tanh(U_a), \tag{0.22}$$

n^a is the average decision of group members a, as shown by (0.22). In addition, U_a's in (0.19) is a linear regression model with parameters $K_{a,b}$'s and p_a's. As shown below, the private incentive component p_a is characterized as a linear regression.

$$p_a = \sum_{j=1}^{w} \tau_j \varphi_a^j + \tau_0 \tag{0.23}$$

where w represents the number of attributes, τ_j's represents the relative weights that women give to their attributes, and τ_0 represents the base private incentive. In our case, $w = 2$. As a result, the parameters that must be estimated are $K_{a,b}$, τ_j and τ_0 [2]. We utilized MATLAB software version R2016a for carrying out our analyses and obtaining results.

Estimation

The least squares method is used to calculate the model parameters. As a result, we must identify the parameter settings that minimizes

$$\sum_a \left[\bar{n}^a - \tanh(U_a)\right]^2 \tag{0.24}$$

where $\bar{n}^a$ is the average choice of group a. Because $\tanh(U_a)$ is non-linear, the computations will take an extremely long time, see [2]. In the interaction scenario, the

independent variables are correlated. As a result, the least squares method is rendered ineffective. In that case, the partial least squares estimation method will be utilized. We would then calculate the weighted averages for the different groups using Tables 2 and 3 (see main manuscript). The weighted average is $n^a = \frac{D_a}{D}\bar{n}^a$ and if $\bar{n}^a = \frac{1}{D_a}\sum_{i\in V_{Da}} \varphi_i$ is substituted into it we get that

$$n^a = \frac{1}{D}\sum_{i\in V_{Da}} \varphi_i = \frac{1}{D}\left(D^a_S - D^a_{SA}\right), \tag{0.25}$$

where D^a_S is the number of women that choose to sleep in a treated bed net in group a and D^a_{SA} is the number of women in group a that do not choose to sleep in a treated bed net, for $a = 1, 2, 3, 4$ and D the total population size. In particular, $a = 1$ represents pregnant women that are anaemic

$$n^1 = \frac{1}{2434}(116 - 21)$$

$$= \frac{95}{2434}$$

$a = 2$ represents pregnant women that are non anaemic

$$n^2 = \frac{1}{2434}(71 - 7)$$

$$= \frac{32}{1217}$$

$a = 3$ represents non-pregnant women that are anaemic

$$n^3 = \frac{1}{2434}(1117 - 147)$$

$$= \frac{951}{2434}$$

$a = 4$ represents non-pregnant women that are non anaemic

$$n^4 = \frac{1}{2434}(789 - 147)$$

$$= \frac{321}{1217}.$$

References

1. Selinger JV. Introduction to the theory of soft matter: from ideal gases to liquid crystals. Berlin, Germany: Springer International Publishing; 2016.
2. Opoku AA, Osabutey G, Kwofie C. Parameter evaluation for a statistical mechanical model for binary choice with social interaction. Journal of Probability and Statistics. 2019 Mar 4;2019.
