# Supplementary material for: The relationship between anaemia and the use of treated bed nets among pregnant and non-pregnant women in Ghana
Source: PLoS One. 2024 May 2;19(5):e0300431. doi: 10.1371/journal.pone.0300431 (PMC11065244; doi:10.1371/journal.pone.0300431)
Supplement: Attachment — Submitted filename: Response to Reviewers.docx [file pone.0300431.s005.docx]

| Journal Requirements: | Response |
| --- | --- |
| Please review your reference list to ensure that it is complete and correct. If you have cited papers that have been retracted, please include the rationale for doing so in the manuscript text, or remove these references and replace them with relevant current references. Any changes to the reference list should be mentioned in the rebuttal letter that accompanies your revised manuscript. If you need to cite a retracted article, indicate the article’s retracted status in the References list and also include a citation and full reference for the retraction notice. | The reference list has been updated: Reference 47, appearing on lines 582-584, has been deleted because it was not used in the paper. This was an oversight. |

| Editor Comments | Response |
| --- | --- |
| 1. Title: The study did not assess nor include any variable called ‘anaemia level’. What was included was the presence or absence of anaemia, i.e., anaemia or not. This is not ‘anaemia level’. Hence, I suggest the title be further modified as ‘The relationship between anaemia and the use of treated bed nets among pregnant and non-pregnant women in…. region of Ghana.’ | Thank you for your comment. Please see the title of the revised manuscript for the updates. |
| 2. Line 218: Authors should insert a sub-heading titled ‘Statistical Analysis.” | Thank you for your comment. Please see line 218 of the revised manuscript for the updates. |
| 3. Line 370: The “Model Diagnostics and Validation” section, placed after the discussion, should be moved to the Methods section under the “Statistical Analysis" sub-section. | Thank you for your comment. Please see lines 314-335 of the revised manuscript for the updates. |
| 4. Lines 229 – 231: This suggests that the variables ‘pregnancy status’ and ‘anaemia’ determined the decision of respondents to use treated nets. This implies a causal relationship which, in terms of health epidemiology, cannot be determined by the cross-sectional data used in the study. It should be stated simply as a relationship between the variables. The same observation applies to statements made in the discussion (lines 317-319 and 326) and conclusion (lines 392-393) | Thank you for your comment. Please see abstract, lines 162-165, lines 337-338, lines 393-395, and 368-369 of the revised manuscript for the updates. |
| 5. Lines 320-323: As the authors rightly observed here, the study's findings should be stated as a statistical relationship between the dependent variable (use of nets) and the covariates. | Thank you for your comment. |
| 6. Tenses of verbs Some tenses still need to be changed to past tests since the study has already been conducted. These include (but are not limited to) the following: Lines 266: “We will get…” Lines 273-274: “We will now compute…” should changed to “we then computed.” Lines 228 – 229: “…will be used” | Thank you for your comment. Please see lines 174-188, lines 231-238, lines 251-262, line 265, and lines 271-276 of the revised manuscript for the updates |
| 7. Conclusion Lines 397-398: The statement here that pregnant and anaemic women had higher usage of bed nets contradicts the statement in lines 405-406, stating that bed net use among the same pregnant and anaemic women is low. | Thank you for your comment. Please see line 404 of the revised manuscript for the updates. |
